# Diversity, Assembly, and Habitat-Driven Dynamics of Microbial Communities in Eutrophic Dianchi Lake, Southwest China

**DOI:** 10.3390/microorganisms14030554

**Published:** 2026-02-28

**Authors:** Jun Chen, Zhizhong Zhang, Bowen Wang, Jiaojiao Yang, Guangxiu Cao, Jinyan Dong, Tao Li, Yanying Guo

**Affiliations:** 1 State Key Laboratory for Conservation and Utilization of Bio-Resources in Yunnan, Yunnan University, Kunming 650091, China; 2 Kunming Dianchi & Plateau Lake Research Institute, Kunming 650228, China

**Keywords:** abundant taxa, rare taxa, microbial communities, spatial heterogeneity, plateau eutrophic lake

## Abstract

Microbial communities are key regulators of ecological processes in aquatic ecosystems and serve as sensitive indicators of environmental change. Here, we investigated the diversity, assembly mechanisms, and spatial differentiation of bacterial and fungal communities across three representative regions of Dianchi Lake—a large, shallow, eutrophic plateau lake in Southwest China characterized by severe nutrient enrichment and organic pollution. The lake was divided into a submerged macrophyte remnant zone (*SubmP*), the heavily polluted Caohai area (*hPollut*), and a cyanobacterial bloom zone (*HABs*). Amplicon sequencing of the 16S rRNA and ITS genes revealed 7862 bacterial and 3141 fungal OTUs, spanning 69 bacterial phyla (1128 genera) and 9 fungal phyla (477 genera). Although 69 dominant bacterial genera (e.g., *Flavobacterium*) and 9 dominant fungal genera (e.g., *Metschnikowia*) were shared across regions, pronounced spatial heterogeneity was observed, primarily driven by total nitrogen and dissolved oxygen. Taxonomic richness and abundance were decoupled: rare (*RT*) and intermediate taxa (*IT*) accounted for the most richness, whereas abundant taxa (*AT*) dominated the total abundance but exhibited comparatively low diversity. *IT* and *RT* displayed significantly higher Shannon diversity and greater network robustness than *AT*; bacterial *RT* showed the highest robustness (0.35–0.45), while fungal *IT* demonstrated superior resilience. Community assembly was largely governed by stochastic processes (59–99% contribution), yet deterministic selection exerted stronger effects on *IT* and *RT*, particularly for bacteria in *SubmP*, where habitat heterogeneity enhanced environmental filtering. Functional prediction revealed distinct ecological strategies, with enhanced nitrogen cycling in *hPollut*, phototrophy in *HABs*, and pollutant degradation in *SubmP*. Collectively, these findings demonstrate that rare and intermediate taxa, rather than numerically dominant populations, underpin microbial stability and spatial differentiation in eutrophic lakes, highlighting the importance of nitrogen management and habitat heterogeneity in lake restoration.

## 1. Introduction

In shallow freshwater ecosystems, aquatic plants (such as emergent, floating-leaved, submerged, and floating plants) are considered ecosystem engineers due to their profound and multifaceted ecological regulatory functions [1]. As integral components, aquatic plants can maintain the functionality of water ecosystems through multiple interacting pathways. Their physical structures modify the surrounding environment by creating heterogeneous habitats that support diverse aquatic communities [2]. For example, the root networks of aquatic plants stabilize sediments, significantly reducing water turbidity, while emergent and submerged canopies generate vertical light gradients that can limit excessive phytoplankton proliferation through shading effects [3]. Such physical heterogeneity provides various microhabitats for aquatic organisms [2]. Beyond their structural roles, aquatic plants actively participate in biogeochemical cycling. By absorbing dissolved nitrogen and phosphorus, they compete with algae for these essential nutrients and help to regulate nutrient availability in the water column [4]. Oxygen released from plant rhizospheres can create localized oxic–anoxic interfaces, potentially enhancing processes such as denitrification [5]. Moreover, the decomposition of plant litter contributes to the maintenance of the carbon–nitrogen cycle balance in the water, supporting ecosystem health and water quality [6]. Aquatic plants also contribute to biodiversity by increasing habitat complexity across trophic levels. Microorganisms can colonize plant surfaces; invertebrates occupy root zones, which serve as refuges; and fish larvae often depend on vegetated canopies for nursery habitats [7,8]. Through these combined structural and functional roles, aquatic vegetation can buffer aquatic systems against eutrophication, hydrological fluctuations, and other stressors by retaining nutrients, attenuating hydrodynamic forces, and influencing sediment oxidation [5,9,10]. Because of these functions, the United Nations Environment Programme has listed aquatic plants as essential indicators in assessing the health of shallow ecosystems, and they have become vital components in ecological restoration and water quality management.

However, owing to multiple negative anthropogenic effects, aquatic plants are experiencing a global decline, leading to a shift toward turbid, algae-dominated states [11]. The resilience of aquatic plant communities has been progressively weakened by multiple interacting stressors. For example, eutrophication stimulates algal blooms that reduce underwater light availability to below the compensation point required by submerged plants [12]. Elevated levels of organic pollutants (such as chemical oxygen demand (COD) > 50 mg/L) can directly impair root metabolism and reduce seedling survival in numerous aquatic macrophyte species [13]. In addition, hydrological alterations such as dam construction and shoreline hardening disrupt the reproductive habitats of emergent plants [14]. Since the twentieth century, these combined pressures have led to a sharp decline in global aquatic plant coverage [11]. More critically, the disappearance of aquatic vegetation can initiate self-reinforcing feedbacks: reduced sediment stabilization increases resuspension, while weakened nutrient competition promotes the dominance of cyanobacteria [15]. Without timely intervention, such shifts are often difficult to reverse, as exemplified by the persistent turbid states observed in Taihu Lake (China) and Lake Apopka (USA) following the collapse of aquatic plants [16,17].

Recent studies emphasize the crucial role of the microbiome associated with aquatic plants in enhancing their hosts’ resistance to environmental stresses such as eutrophication, algal blooms, organic pollutants, and light attenuation [18]. In eutrophic waters, the rhizosphere microbiota helps mitigate plant nutrient stress by regulating nitrogen and phosphorus acquisition and balancing the root environment. These microorganisms mediate processes such as nitrification–denitrification and phosphorus immobilization, thereby alleviating nutrient toxicity and promoting a more balanced nutrient supply for their host plants [19,20]. During blue–green algal blooms, certain microbes, such as *Streptomyces* sp. TH05, disrupt cyanobacterial cells and inhibit the growth of *Microcystis aeruginosa* through extracellular bioactive compounds that damage the cell membrane, thereby maintaining the leaf surface light intensity at optimal photosynthetic levels [21]. Under low-light conditions, some photosynthetic bacteria (e.g., *Rhodopseudomonas*) associated with the plant canopy may help submerged macrophytes maintain photosynthetic efficiency [22]. In organic-polluted waters, microorganisms degrade pollutants such as chlorobenzene and polycyclic aromatic hydrocarbons around plant tissues, reducing pollutant toxicity [23,24]. Furthermore, microorganisms activate compounds associated with plant systemic resistance, such as *Bacillus*-produced a chlorophyll-c (ACC) deaminase, which lowers ethylene levels and delays leaf senescence under low-light stress [25]. Therefore, the “plant–microbe” synergistic resistance system is essential in maintaining the stability of vegetation communities in degraded water ecosystems [18], and ecological restoration through plant–microbe interactions offers a feasible and sustainable approach [26].

Dianchi Lake, a typical large, shallow lake in the Yunnan–Guizhou Plateau (approximately 300 km^2^ in area, with an average depth of 5.3 m), is inherently fragile due to its semi-closed basin and hydraulic residence time of 4–5 years [27]. The lake has long been under pressure from urban sewage, industrial wastewater, and agricultural non-point-source pollution due to its proximity to the city of Kunming (with a population exceeding 8 million) [27]. In terms of the evolution of the aquatic vegetation community, Dianchi Lake has experienced a pattern of structural integrity followed by severe degradation [28]. Currently, the lake exhibits clear spatial variation: the northern Caohai area continues to experience high organic pollution, and the central and southern outer lake areas are dominated by algal blooms (chlorophyll-a > 50 μg/L), while the western lakeshore’s emergent plant remnant zone has become a key area for lake ecological restoration [29]. Based on this spatial differentiation, the present study focuses on three representative regions: Caohai in the north, the outer lake zone with algal blooms in the center and south, and the western lakeshore’s emergent plant remnant zone. The compositions and succession patterns of the microbial communities in these areas are systematically evaluated. The aim is to elucidate the microbial community’s response under varying pollution gradients, providing scientific support for the ecological restoration of Dianchi Lake.

## 2. Materials and Methods

### 2.1. Study Site and Sample Collection

**Figure 1:**
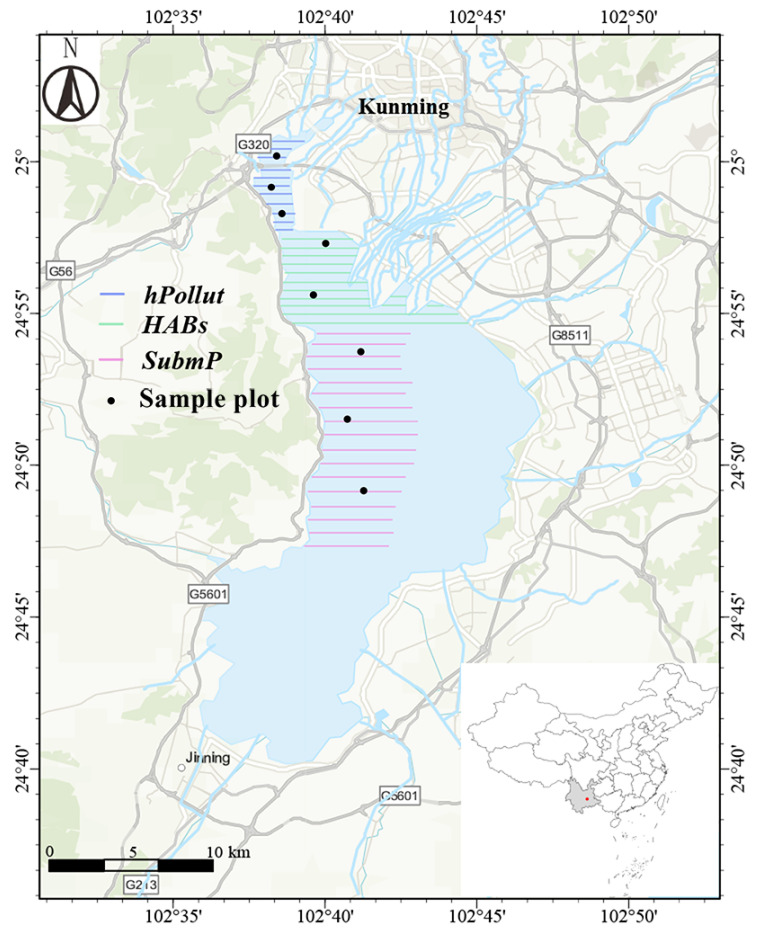
Sampling site map of the three representative regions of Dianchi Lake: the heavily polluted Caohai area (*hPollut*), the cyanobacterial bloom zone (*HABs*), and the submerged macrophyte remnant area (*SubmP*).

Dianchi Lake (24°40′–25°02′ N, 102°36′–102°47′ E) is a large, shallow, semi-enclosed lake located in the city of Kunming, Yunnan Province, Southwest China. The lake surface has an elevation of 1887.5 m, with an area of 309.5 km^2^ and an average depth of 5.3 m. Since the construction of a human-made barrier in 1996, the lake has been hydrologically divided into two distinct parts: Caohai (with an area of 10.8 km^2^ and an average depth of 2.3 m) and the outer lake (the main basin, with an area of 298.7 km^2^ and an average depth of 5.3 m) [30]. In this study, three representative areas were selected: the heavily polluted Caohai area (*hPollut*), the algal bloom aggregation area in the outer lake (*HABs*), and the submerged macrophyte remnant area (*SubmP*) (Figure 1). The *hPollut* zone, located to the north of the sightseeing embankment, historically served as the primary receiving basin for most urban pollutants discharged from the city of Kunming prior to large-scale restoration efforts in Dianchi. The water body is dominated by planktonic algae accumulations, particularly cyanobacteria such as *Microcystis* spp. [31]. The *HABs* zone lacks visible submerged vegetation and is influenced by the southwest prevailing winds and lake currents, making it a key area for sustained algal accumulation. The peak cyanobacterial bloom typically occurs from May to November each year [32]. The *SubmP* zone is characterized by high species diversity and substantial plant coverage, reaching up to 10%. Dominant species include *Stuckenia pectinata*, *Potamogeton wrightii*, *Hydrilla verticillata*, and *Myriophyllum spicatum* [33]. Additionally, this region has historically been free of direct industrial pollution and is less populated compared to other areas of Dianchi Lake—particularly the eastern region near the main urban area and the heavily polluted Caohai. The relatively low external pollution load in this area creates a favorable environment for the stable growth of submerged vegetation.

Three representative regions of the lake (*hPollut*, *HABs*, and *SubmP*) were selected as study sites. Three independent sampling plots were examined for each region, except for the *HABs* site, where one sampling plot was excluded, as it did not meet the quality control requirements during sample preservation, leaving two plots for investigation. Water sampling was conducted on 16 July and 15 October 2024, with three biological replicates collected from each plot at each sampling time point. In total, 48 water samples were obtained, calculated as (2 + 3 + 3) plots × 3 biological replicates × 2 sampling periods. For each individual sample, 1000 mL of surface water was collected at a depth of 0.5 m using a water sampler (Global Shangqing Technology Co., Ltd., Beijing, China). Immediately after collection, samples were stored in a cooler at 4 °C and transported to the laboratory within 12 h for further processing. In the laboratory, each water sample was divided into two subsamples: one for microbial analysis and the other for physicochemical measurements. For microbial analysis, water samples were pre-filtered to remove large particulate matter and subsequently passed through sterile 0.22 μm membrane filters. The filters were then stored at −80 °C until further analysis. During sampling, the water temperature, dissolved oxygen, and electrical conductivity were measured in situ at each sampling site using a multiparameter probe (YSI 660, Yellow Springs Instruments, Yellow Springs, OH, USA). Water transparency was additionally determined using a Secchi disk (SD20, Beijing Purity Instrument Co., Ltd., Beijing, China).

### 2.2. Physicochemical Properties of Water Samples

Total nitrogen (TN) in water was quantified using the sulfate potassium oxidation–ultraviolet double-wavelength calibration method, according to Chinese national environmental standards (HJ 636-2012). Total phosphorus (TP) was measured by ammonium molybdate spectrophotometry at 700 nm (GB/T 11893–1989). Chlorophyll-a (Chl-a) was measured using the four-wavelength matrix algorithm (750/664/647/630 nm) (HJ 897-2017). pH was determined using a portable pH meter (PHB-4, INASE Scientific Instrument Co., Ltd., Shanghai, China) in accordance with national standards (HJ 1147-2020). Chemical oxygen demand (COD) was determined by the potassium dichromate method (HJ 828-2017), and suspended substances (SS) were measured using the gravimetric method (GB 11901-89). Biochemical oxygen demand (BOD_5_) was determined using the dilution and seeding method (HJ 505-2009). The permanganate index was measured via the acid potassium permanganate method (GB 11892-89).

### 2.3. Analysis of Microbial Community Diversity in Water Samples

The filter membranes containing bacteria and fungi were cut into pieces, and total DNA was extracted using the PowerSoil^®^ DNA Isolation Kit (MoBio, Carlsbad, CA, USA). The DNA concentration and quality were measured using a Thermo NanoDrop 2000 UV spectrophotometer (Thermo Scientific, Waltham, MA, USA) and verified by 1% agarose gel electrophoresis. After quality inspection, the DNA was amplified using bacterial 16S rDNA universal primers 515F (5′-GTG CCA GCM GCC GCG G-3′) and 907R (5′-CCG TAA TTC MTT TRA GTT T-3′) and fungal ITS universal primers ITS1F (5′-CTT GGT CAT TTA GAG GAA GTA A-3′) and ITS2R (5′-GCT GCG TTC TTC ATC GAT GC-3′) [34]. Fungal amplification was carried out using a 20 μL reaction mixture containing 10 μL of 10× buffer, 2 μL of 2.5 mM dNTPs, 0.8 μL of each primer (5 μM), 0.2 μL of rTaq Polymerase (TaKaRa rTaq DNA Polymerase), 0.2 μL of BSA, and 10 ng of DNA template. Bacterial amplification was performed using 4 μL of 5×FastPfu Buffer, 2 μL of 2.5 mM dNTPs, 0.8 μL of each primer (5 μM), 0.4 μL of FastPfu Polymerase (TransGen AP221-02: TransStart Fastpfu DNA Polymerase), 0.2 μL of BSA, and 10 ng of DNA template. The PCR program consisted of initial denaturation at 95 °C for 3 min, followed by 27 cycles of 95 °C for 30 s, 55 °C for 30 s, and 72 °C for 45 s, with a final extension at 72 °C for 10 min. Products were detected by 2% agarose gel electrophoresis, purified using the Trelief^®^ Gel DNA Recovery Kit (Tsingke Biotechnology Co., Ltd., Beijing, China), and quantified using a DS-11 spectrophotometer (DeNovix, Wilmington, DE, USA). After library construction with the NEXTflex Kit (Bio Scientific, Austin, TX, USA), paired-end sequencing was performed on the Illumina NextSeq 2000 platform (Illumina, San Diego, CA, USA).

Raw data were filtered using Fastp (v0.19.6) software to remove low-quality reads (Q < 20), and high-quality sequences were assembled and optimized using FLASH (v1.2.11). OTUs were clustered at a 97% sequence similarity threshold based on the UPARSE (v7.1) algorithm [35]. The taxonomic annotation of OTUs was performed using the RDP classifier (v2.11) combined with the UNITE (v8.0) database, with a confidence threshold of ≥97%, and the abundance of each taxonomic unit was calculated [36]. A series of microbial diversity analyses, including α-diversity, β-diversity, species composition, species differential comparison, environmental factor correlation, and bacterial–fungal interaction network analysis, were performed on the Majorbio Cloud Platform (https://www.majorbio.com/tools, accessed on 1 October 2025) based on the normalized OTU table. To characterize ecological functions, we employed FAPROTAX for bacterial annotation and FUNGuild for fungal guild assignment [37,38]. To assess the stability of bacterial and fungal interaction networks in each region, the robustness was calculated by randomly removing 50% of the nodes and determining the proportion of remaining species in the network [39]. Indicator species were selected based on IndVal > 0.7 and *p* < 0.05 [40]. Abundant and rare OTUs were defined following a previously established framework that integrated both local and regional relative abundances [41]. Briefly, OTUs were classified as abundant taxa (*AT*) if their relative abundance within a sample exceeded 1% and their mean relative abundance across all samples was ≥0.1%. OTUs were classified as rare taxa (*RT*) if their relative abundance at the local level was <0.01% and their mean relative abundance across all samples was <0.001%. The remaining OTUs were defined as intermediate taxa (*IT*). Downstream analyses were conducted at three levels: all OTUs, abundant taxa, and rare taxa. The raw sequence data have been deposited in the NCBI SRA under BioProjects PRJNA1346230 and PRJNA1345722.

### 2.4. Microbial Community Assembly Processes in Water Samples

The community assembly processes of bacterial and fungal taxa, as well as the abundant (*AT*), intermediate (*IT*), and rare (*RT*) taxa, in the three representative areas of Dianchi Lake were quantified using a null model analysis [42]. Community assembly processes were classified as deterministic (e.g., heterogeneous or homogeneous selection) or stochastic (e.g., dispersal limitation, homogenizing dispersal, and ecological drift processes) based on a null model framework. The β-nearest taxon index (βNTI) was used to assess phylogenetic turnover by comparing the observed β-mean nearest taxon distance (βMNTD) values to null expectations, thereby inferring the role of deterministic processes. Values of |βNTI| > 2 indicate deterministic assembly, with βNTI > 2 representing heterogeneous selection and βNTI < −2 indicating homogeneous selection. When |βNTI| < 2, stochastic processes are considered dominant. In this case, the Raup–Crick metric based on the Bray–Curtis dissimilarity (RCbray), which is used to evaluate deviations in taxonomic turnover from null expectations, was used to further partition stochastic mechanisms. RCbray > 0.95 indicates dispersal limitation, RCbray < −0.95 indicates homogenizing dispersal, and values between −0.95 and 0.95 suggest ecological drift [42].

### 2.5. Statistical Analysis

Statistical analyses were performed using R (v4.3.2), and data visualization was conducted using Origin 2021 software (Electronic Arts Inc., Northampton, MA, USA). One-way analysis of variance (ANOVA) was used to compare the physicochemical properties of water, Shannon indices of groups with different abundances, and the stability of interaction networks across the three representative areas. All data are presented as the mean ± standard error (SE).

## 3. Results

### 3.1. Physicochemical Properties of Water Samples

The water quality parameters in all three surveyed areas of Dianchi Lake indicated significant eutrophication, with organic pollution and elevated total nitrogen as primary concerns. Total phosphorus (TP) and total nitrogen (TN) concentrations ranged from 0.10 to 0.13 mg/L and 1.9 to 3.2 mg/L, respectively, both exceeding the Class IV water quality limits stipulated in the Environmental Quality Standards for Surface Water (GB 3838-2002). Organic pollution was similarly high, as evidenced by BOD_5_ and permanganate index values exceeding Class III limits. Chemical oxygen demand (COD) ranged from 36.2 to 42.5 mg/L, exceeding the Class V standard by 1.06 to 1.4 times in the *HABs* and *SubmP* areas (Table 1). Chlorophyll-a concentrations ranged from 65.9 to 94.1 μg/L, suggesting the proliferation of algae and further confirming the water’s eutrophication status (Table 1). Additionally, significant spatial heterogeneity in water quality was observed across the three representative areas. The *SubmP* area showed the best water quality, followed by the *HABs* area, while the *hPollut* area had the worst water quality. For example, out of the 12 water quality indicators measured, the *hPollut* area exhibited the maximum values for seven indicators—particularly for TN, TP, chlorophyll-a, and dissolved oxygen, which were all significantly higher than in the other two areas. However, COD and electrical conductivity in the *hPollut* area were significantly lower. In contrast, the *SubmP* area exhibited the lowest values for seven indicators, but its COD was significantly higher than at the other two sites (Table 1).

### 3.2. Community Composition Analysis

**Figure 2:**
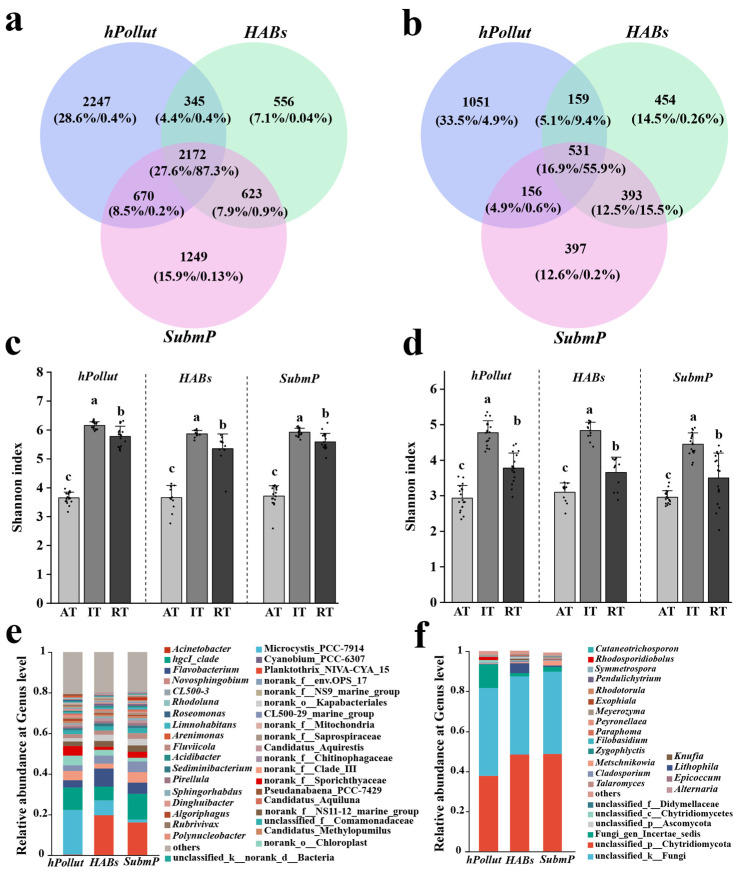
Venn diagrams, Shannon diversity indices, and taxonomic compositions of bacterial (**a**,**c**,**e**) and fungal (**b**,**d**,**f**) communities in water samples from three representative regions of Dianchi Lake: the heavily polluted Caohai area (*hPollut*), the cyanobacterial bloom zone (*HABs*), and the submerged macrophyte remnant area (*SubmP*). “Abundant” (*AT*), “intermediate” (*IT*), and “rare” (*RT*) refer to OTUs with relative abundances > 0.1%, 0.1–0.001%, and <0.001%, respectively. Different lowercase letters indicate statistically significant differences in panels (**c**,**d**) (one-way ANOVA followed by Tukey’s HSD test, *p* < 0.05).

Venn diagram analysis revealed a total of 7862 bacterial OTUs and 3141 fungal OTUs across the three representative regions. The distribution of OTUs varied among the studied regions, with bacterial OTUs ranging from 3696 (26.7%) in the *HABs* region to 5434 (39.3%) in the *hPollut* region (Figure 2a). In contrast, fungal OTUs were most abundant in *hPollut* (1879, 38.7%), while *SubmP* had the fewest (1477, 30.1%) (Figure 2b). Of these OTUs, only 27.6% of bacterial OTUs (2172) and 16.9% of fungal OTUs (531) were shared across all three regions. However, these shared OTUs accounted for 87.3% of the total bacterial relative abundance and 55.9% of the total fungal relative abundance. Conversely, each region contained a considerable number of region-specific OTUs, with bacterial OTU proportions ranging from 7.1% in *HABs* to 28.6% in *hPollut* and fungal OTU proportions ranging from 12.6% in *SubmP* to 33.5% in *hPollut*. Despite the large number of region-specific OTUs, their total relative abundances were all below 0.4%, with the exception of *hPollut*-specific fungal OTUs, which contributed 4.9%. Furthermore, the bacterial communities across the three regions were predominantly composed of rare taxa (*RT*), with the proportion of *RT* OTUs exceeding 57.9% in each region. Intermediate taxa (*IT*) contributed between 27.9% and 38.4%, while abundant taxa (*AT*) made up less than 3.8%. However, in terms of relative abundance, there was a notable contrast between these groups: *RT* taxa contributed less than 1.2% of the total relative abundance, while *AT* taxa accounted for more than 82.5%. Similarly, in fungal communities, clear decoupling between OTU richness and relative abundance was observed: abundant taxa (comprising <7.2% of OTUs) contributed over 89.4% of the abundance, whereas rare taxa (comprising >31.8% of OTUs) accounted for less than 0.35%. In contrast to the most abundant bacterial *AT*, the fungal community was dominated by intermediate taxa (*IT*), which represented over 47.6% of the fungal OTUs in terms of richness. Rare taxa (*RT*) comprised <45.2% of the fungal OTUs, while abundant taxa (*AT*) made up less than 7.2% of the richness (Figure 2a,b). Further analysis of the shared OTUs revealed that intermediate taxa (*IT*) were the most dominant, accounting for over 53.1% of the shared bacterial and fungal OTUs. In contrast, for region-specific OTUs, bacterial communities were dominated by rare taxa (>91.7% of OTUs), while fungal communities showed a more balanced distribution between intermediate (*IT*) and rare taxa (*RT*), with the exception of the significantly higher numbers in *SubmP*. These findings indicate considerable spatial differentiation within the microbial communities of Dianchi Lake. While no significant spatial variation in the Shannon diversity index was observed across the three studied areas for either bacteria (4.50–4.55) or fungi (3.37–3.59), a clear pattern emerged when taxa were classified by their abundance. Intermediate taxa (*IT*) consistently exhibited the highest Shannon diversity, reflecting their key role as the primary contributors to overall microbial diversity. Rare taxa (*RT*) followed, while abundant taxa (*AT*) showed the lowest diversity indices (Figure 2c,d).

A total of 69 bacterial phyla and 1128 genera, as well as 9 fungal phyla and 477 genera, were annotated across the three representative regions, including 127 dominant bacterial genera and 25 dominant fungal genera with abundance > 0.1%. The number of dominant bacterial phyla ranged from 15 (*HABs*) to 16 (*hPollut* and *SubmP*), with 15 phyla, including Actinomycetota, Pseudomonadota, and Cyanobacteriota, shared across all three regions. The number of dominant bacterial genera ranged from 95 (*SubmP*) to 100 (*HABs*), with 68 genera, including *Planktothrix_NIVA-CYA_15*, *Microcystis_PCC-7914*, and *Flavobacterium*, shared by all three regions. Moreover, *hPollut* had 17 region-specific dominant bacterial genera—including *Pseudanabaena*_PCC-7429, *Brevundimonas*, and *Dolichospermum*_NIES41—significantly more than in *HABs* and *SubmP* (which had only 4 each) (Figure 2e). Five dominant fungal phyla, including Chytridiomycota and Ascomycota, were detected across all regions. The number of dominant fungal genera ranged from 14 in *hPollut* to 18 in *SubmP*, with nine genera shared across all regions, including *Fungi_gen_Incertae_sedis*, *Lithophila*, *Metschnikowia*, and *Cladosporium*. Additionally, *hPollut* and *SubmP* each had three region-specific dominant fungal genera, including *Zygophlyctis* in *hPollut* and *Filobasidium* in *SubmP*. Finally, *HABs* had fourunique dominant fungal genera, such as *Pendulichytrium* (Figure 2f).

### 3.3. Community Composition Differences

**Figure 3:**
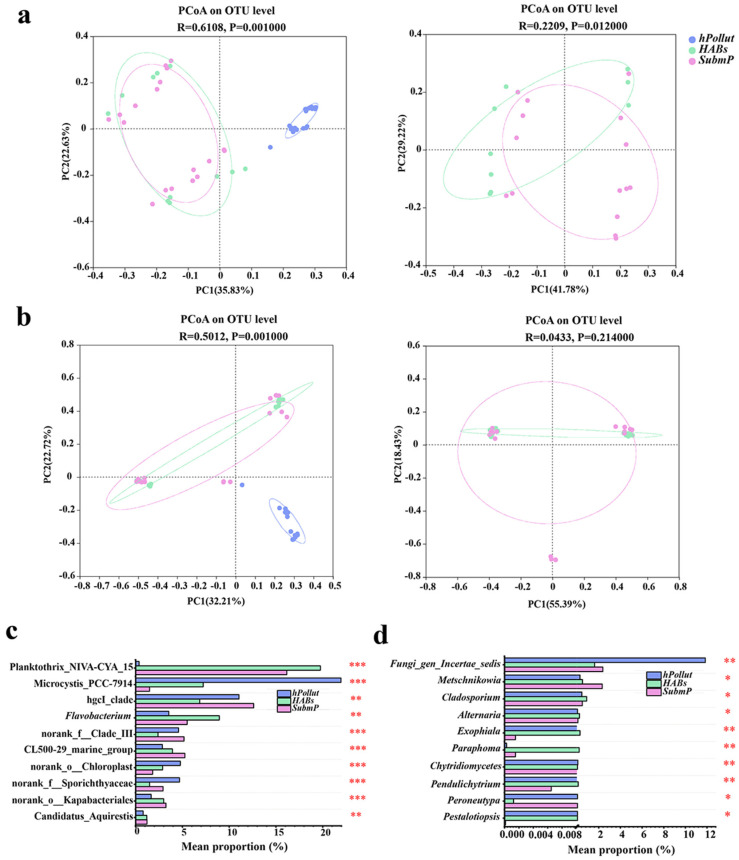
Principal coordinate analysis (PCoA) and differential abundance analysis of bacterial (**a**,**c**) and fungal (**b**,**d**) communities in water samples from three representative regions of Dianchi Lake: *hPollut*, *HABs*, and *SubmP*. (*, *p* < 0.05; **, *p* < 0.01; ***, *p* < 0.001; Top 10).

Principal coordinate analysis (PCoA) revealed significant differences in the bacterial and fungal communities across the three representative regions, except for the fungal communities between *HABs* and *SubmP*, which showed no significant differences (Figure 3a,b). Differential community composition analysis confirmed these findings, revealing that 28.5% (321/1128) of bacterial genera and 7.9% (38/477) of fungal genera showed significant differences in abundance (*p* < 0.05, Kruskal–Wallis H test). Notably, some shared dominant taxa exhibited significant changes in abundance among regions. For example, the bacterial genus *Microcystis_PCC-7914* and the fungal genus *Fungi_gen_Incertae_sedis* were significantly enriched in *hPollut*, with relative abundances of 22.6% and 11.84%, respectively, compared to 6.3% and 2.74% in *HABs*; these values were even lower in *SubmP* (1.3% and 2.36%). Conversely, bacterial *Planktothrix_NIVA-CYA_15* and fungal *Lithophila* were less abundant in *hPollut* (0.36% and 0.16%, respectively) but significantly more abundant in *SubmP* (16.16% and 4.94%) and *HABs* (19.75% and 0.69%). Additionally, the fungal genus *Rhodosporidiobolus* had a relative abundance of 1.47% in *hPollut* but only 0.002% in *HABs*, indicating its rarity (Figure 3c,d).

### 3.4. Prediction of Microbial Function

**Figure 4:**
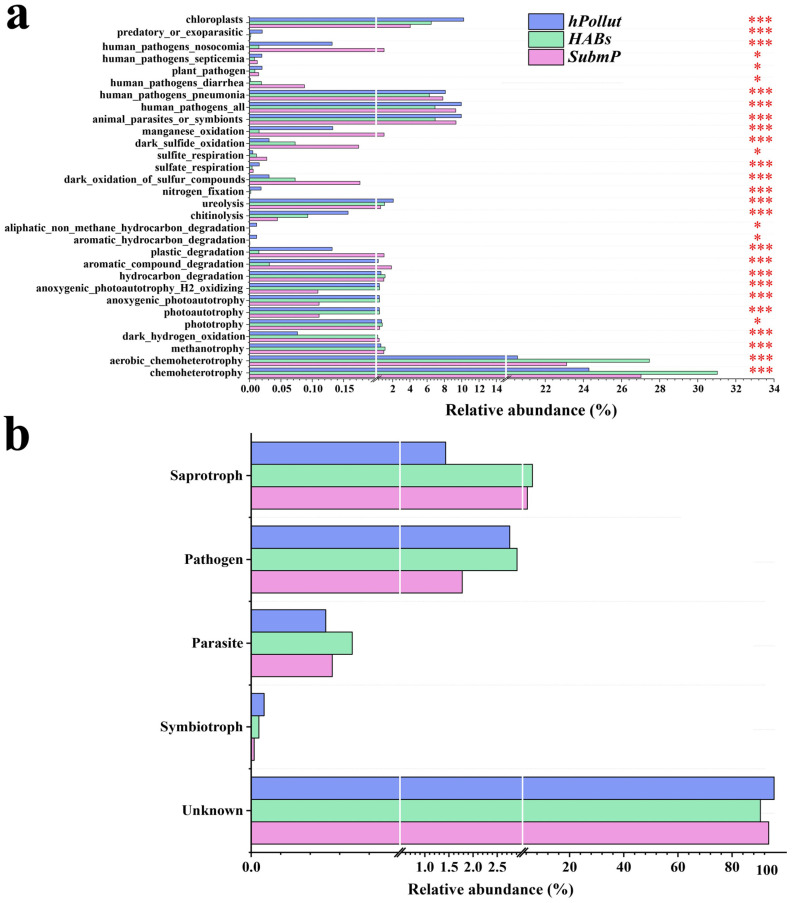
Functional predictions of bacterial (**a**) and fungal (**b**) communities in water samples from three representative regions of Dianchi Lake: *hPollut*, *HABs*, and *SubmP*. (*, *p* < 0.05; ***, *p* < 0.001; Kruskal–Wallis H test).

A total of 68 functional groups were annotated for bacteria in the FAPROTAX database, with 30 groups (44.1%) showing significant differences (*p* < 0.05). Of these, 6.7% (2/30) were related to nitrogen metabolism, including nitrogen fixation and urea degradation; 43.3% (13/30) were associated with carbon cycling; 13.3% (4/30) with sulfur cycling; and 26.7% (8/30) with pathogens. Significant functional differentiation was observed among the microbial communities in the three regions in terms of metabolic potential and ecological function. *hPollut* was enriched with functions related to human health (e.g., human_pathogens_all, pneumonia, septicemia), plant diseases (plant_pathogen), nitrogen cycling (nitrogen_fixation, ureolysis), organic pollutant degradation (aromatic_hydrocarbon_degradation), and predation and symbiosis (predatory_or_exoparasitic, animal_parasites_or_symbionts), reflecting its severe eutrophication, frequent algal blooms, and potential public health risks. *HABs* primarily exhibited functions related to carbon cycling and photosynthesis, such as hydrocarbon_degradation, aerobic_chemoheterotrophy, methanotrophy, and anoxygenic_photoautotrophy, indicating that communities in this region primarily engage in organic matter degradation and light energy utilization. *SubmP* was enriched with functions related to reductive metabolism and pollutant treatment, such as manganese_oxidation, sulfite_respiration, plastic_degradation, and dark_sulfide_oxidation, suggesting that its microbial communities possess strong capacities for pollutant transformation and degradation. Overall, the microbial communities in the three regions exhibited distinct ecological differentiation in terms of functional structure, further corroborating the physicochemical results regarding water quality (Figure 4a). Based on FUNGuild-predicted functional classification, fungal communities were primarily assigned to saprotrophs, pathogens, parasites, and symbiotrophic guilds. Pathogenic fungi were primarily enriched in *hPollut* and *HABs*, while *SubmP* was dominated by saprotrophic fungi (Figure 4b).

### 3.5. Microbial Interaction Networks and Indicator Species

**Figure 5:**
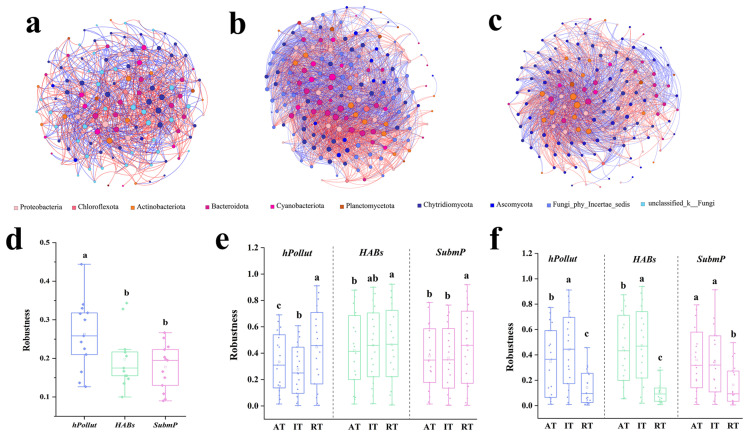
Co-occurrence interaction networks (**a**–**c**) (Top 100) and robustness (**d**–**f**) of bacterial and fungal communities in water samples from three representative regions of Dianchi Lake: *hPollut* (**a**), *HABs* (**b**), and *SubmP* (**c**). Red and blue edges indicate positive and negative correlations, respectively. Different letters indicate significant differences (*p* < 0.05, one-way ANOVA).

The co-occurrence network analysis revealed that all three regions had relatively complex fungal and bacterial interaction networks, with modularity coefficients ranging from 0.231 to 0.317. Positive correlations dominated the bacterial–fungal interactions in all three regions. Moreover, the 20 core nodes with the highest connectivity were region-specific, with no core nodes shared across all regions. The core nodes with the highest connectivity in *hPollut*, *HABs*, and *SubmP* were BOTU24013 (Pseudomonadota), BOTU3684 (Cyanobacteria), and BOTU6784 (Actinobacteriota), respectively. The microbial network stability analysis showed that, after randomly removing 50% of the OTUs, robustness stability ranged from 0.1 to 0.45, with *hPollut* exhibiting the highest value, being significantly higher than in the other two regions (Figure 5, Table S1). A clear pattern also emerged when taxa were categorized by abundance. For bacteria, the subcommunity of rare taxa (*RT*) exhibited the highest robustness, being significantly greater than that of both the intermediate (*IT*) and abundant (*AT*) groups. In contrast, the *IT* subcommunity showed the lowest robustness, being significantly lower than that of both *RT* and *AT*. For fungi, however, the *IT* subcommunity displayed the highest robustness, being significantly greater than that of *RT* and *AT*, while the *RT* subcommunity had the lowest robustness (Figure 5, Table S1). These findings highlight the contrasting responses of bacterial and fungal communities to environmental disturbances across different regions.

The indicator species analysis revealed 1159 bacterial OTUs and 452 fungal OTUs across the three regions; more than 70.1% of these were associated with intermediate taxa (*IT*). Among these, 133 bacterial and 48 fungal OTUs were classified as strong indicators (*IndVal* > 0.7, *p* < 0.05). Notably, the *hPollut* region harbored the highest number of strong indicator species, including 126 bacterial and 47 fungal OTUs, suggesting a distinct and highly specialized microbial assemblage in this area (Table S2).

### 3.6. Analysis of Physicochemical Properties and Microbial Correlations

**Figure 6:**
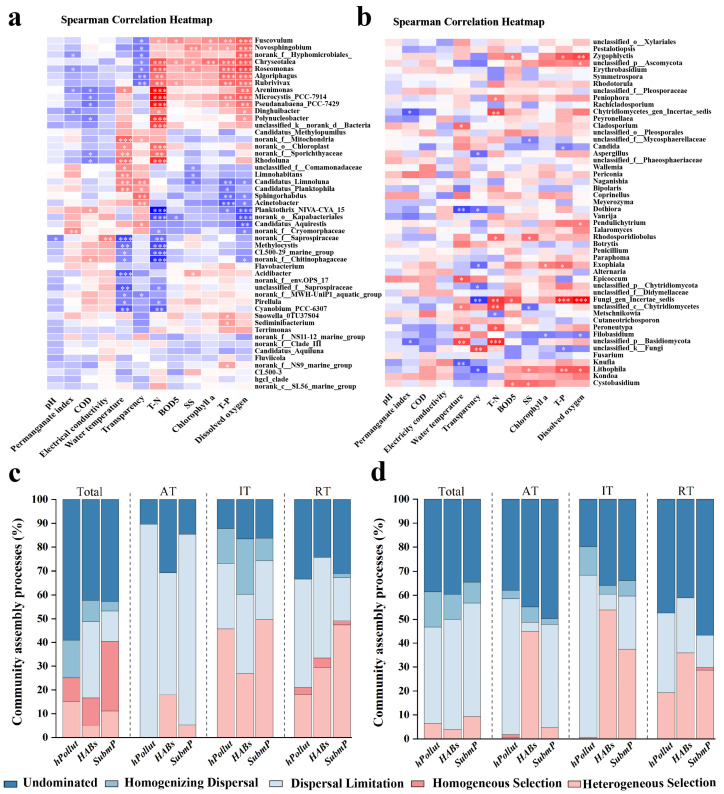
Spearman correlation heatmap between environmental parameters and dominant bacterial (**a**) and fungal (**b**) taxa (Top 50) and community assembly processes of bacterial (**c**) and fungal (**d**) communities in water samples from three representative regions of Dianchi Lake: *hPollut*, *HABs*, and *SubmP*. (* *p* < 0.05; ** *p* < 0.01; *** *p* < 0.001; Wilcoxon rank-sum test; red indicates deterministic processes, while blue represents stochastic processes).

Correlation analysis of the 50 most abundant bacterial and fungal species and water physicochemical factors showed that total nitrogen (TN) was the greatest influencing factor for both bacteria (48% of species) and fungi (16% of species). Dissolved oxygen (DO) was the second-greatest influencing factor for bacteria, while temperature and transparency had the second-greatest influences for fungi. Surprisingly, COD was not a major factor affecting the microbial communities in Dianchi Lake, showing no significant correlation with the top 50 fungal species (Figure 6a,b). Moreover, the bacterial genus *Chryseotalea* and the fungal genus *Fungi_gen_Incertae_sedis* were the most sensitive to changes in water quality parameters; their levels were significantly correlated with seven and five water physicochemical properties, respectively (Figure 6a,b).

### 3.7. Community Assembly Processes Results

Comparison of the community assembly processes across the three regions showed that both bacterial and fungal communities were predominantly shaped by stochastic processes, which accounted for more than 59% of bacterial assembly and over 90% of fungal assembly. However, the bacterial and fungal communities exhibited different assembly characteristics depending on their microbial types, spatial distributions, and microbial abundances. For example, compared with the *IT* and *RT* groups, the *AT* group in both the bacterial and fungal communities showed a marked increase in the contribution of stochastic processes, with the proportion of stochastic assembly in bacteria reaching 99.4% in the *hPollut* region (Figure 6c,d).

## 4. Discussion

### 4.1. Spatial Heterogeneity of Microbial Communities in Dianchi Lake and Their Driving Factors

This study revealed pronounced differences in the microbial communities across three representative regions of Dianchi Lake, highlighting its strong ecological heterogeneity. Previous studies have similarly reported that the microbial communities in aquatic systems are highly responsive to environmental heterogeneity [43]. The core drivers of microbial divergence across lake regions may include organic matter, available phosphorus, pH, and the regulatory effects of submerged macrophytes [44]. Our findings revealed that total nitrogen (TN) was the most influential environmental factor shaping the structure and function of the microbial community, with significant correlations observed in nearly half of the bacterial and fungal OTUs. This aligns well with the “nitrogen dominance” theory, which posits that total nitrogen serves as a major driver of microbial community dynamics in nitrogen-enriched aquatic systems [45]. Ecologically, the availability of various forms of nitrogen (e.g., NO_3_^−^, NH_4_^+^) directly influences key nitrogen-cycling guilds, such as nitrifiers, denitrifiers, and diazotrophs. Meanwhile, elevated TN also promotes algal blooms and subsequent organic matter deposition, shifting redox conditions and indirectly influencing non-nitrogen functional groups [46]. More importantly, the input of nutrients such as nitrogen can lead to eutrophication, triggering cascade effects in the environment that further exacerbate the impacts on microbial community succession in aquatic ecosystems. For example, nitrogen-induced eutrophication can lead to algal overgrowth, followed by decomposition, which consumes dissolved oxygen. This finding is consistent with our study, in which we also identified dissolved oxygen (DO) as another key environmental factor influencing microbial community succession in aquatic ecosystems. Furthermore, nitrogen-induced eutrophication and subsequent algal blooms can alter water transparency, impacting the survival of aquatic plants and their associated microbial communities [47]. Our results suggest that such interactions are a key factor in maintaining the unique microbial community structures across different water regions. Overall, nutrient inputs—particularly nitrogen—appear to be among the most influential factors driving microbial community succession in aquatic ecosystems.

### 4.2. Dominant Genera and Their Ecological Significance

Several dominant genera were consistently detected across all three regions, including *Flavobacterium* and *Metschnikowia*. Although individual OTUs within these genera accounted for relatively small proportions (<27.6%), their combined relative abundance exceeded 55.9%, indicating a clear niche dominance in both the bacterial and fungal communities. These core taxa generally exhibit broad habitat ranges. For instance, *Flavobacterium* is not only abundant in both freshwater and marine ecosystems but also widely reported in diverse habitats, such as sludge, polar permafrost, temperate forest soils, and plant rhizospheres, as well as in stressful environments such as heavy metal-polluted wastewater and petroleum hydrocarbon-contaminated systems [48,49,50,51]. Similarly, the fungal genus *Metschnikowia* is globally distributed across a wide range of environmental settings [52]. Collectively, these distribution patterns suggest that the core microbial taxa in Dianchi Lake possess strong environmental adaptability and tolerance to stressed aquatic conditions. Beyond their broad ecological distributions, these taxa are also associated with multiple functional traits that are relevant to ecosystem processes and environmental resilience. Bacterial members of the genus *Flavobacterium* are known for their ability to degrade a variety of organic pollutants, including petroleum hydrocarbons, highlighting their potential roles in pollutant attenuation and environmental restoration in aquatic and sedimentary systems [48,49]. Additionally, some *Flavobacterium* bacterial species form symbiotic relationships with aquatic plants, exhibiting notable plant-growth-promoting traits and enhancing the health and stress resistance of the host plants [51]. Some *Flavobacterium* strains, such as *Flavobacterium* sp. 5N-3, have demonstrated strong inhibitory activity against the growth of the red-tide-forming alga *Gymnodinium nagasakiense*, particularly during its logarithmic growth phase [53]. Taken together, these shared core microbial taxa are implicated in multiple ecological functions. Their consistent occurrence and functional versatility underscore their potential importance in maintaining ecosystem stability and supporting environmental restoration, warranting further investigation into their specific roles in eutrophic water systems.

### 4.3. Ecological Roles of Intermediate and Rare Taxa in Microbial Networks of Dianchi Lake

Our results indicate that intermediate (*IT*) and rare taxa (*RT*) contribute disproportionately to the microbial community structure in Dianchi Lake, as reflected by their higher Shannon diversity and greater network robustness compared with abundant taxa (*AT*). Similar patterns have been reported in freshwater systems, where *IT* and *RT* taxa represent a substantial fraction of the taxonomic diversity despite their limited numerical dominance [54]. The elevated diversity of *IT* and *RT* likely reflects niche partitioning within heterogeneous aquatic environments. Freshwater lakes often exhibit strong gradients of nutrients, oxygen, and redox conditions, allowing *IT* and *RT* taxa to occupy specialized ecological niches [55,56]. The “rare biosphere” concept, originally proposed in marine and aquatic systems, suggests that these taxa form a reservoir of genetic and functional potential that can influence community dynamics when environmental conditions shift [57]. In lake ecosystems, both *IT* and *RT* have been shown to respond sensitively to environmental perturbations and may increase in abundance under changing conditions, thereby contributing to community turnover [58]. Such dynamics can generate “insurance effects,” whereby functionally similar taxa compensate for declines in dominant populations and help sustain key ecosystem processes [59,60]. The greater robustness observed in the *IT* and *RT* subnetworks further suggests a stabilizing role in microbial interactions. In aquatic microbial communities, complex co-occurrence networks are often associated with higher resistance to disturbances and greater ecological stability [61]. Functional redundancy among *IT* and *RT* taxa may buffer ecosystem processes when dominant groups decline, particularly in eutrophic lakes, where nutrient enrichment and bloom dynamics generate recurrent disturbances. This pattern was especially pronounced in the submerged macrophyte remnant area zone (*SubmP*), where habitat heterogeneity likely supported a broader spectrum of *IT* and *RT* taxa. Aquatic vegetation can create microhabitats with variable oxygen and nutrient conditions, promoting microbial diversification and stabilizing the community structure [62]. Taken together, our findings support the view that the ecological potential of these intermediate and rare taxa enables a more flexible and responsive taxonomic composition, reducing ecosystem lag and promoting the recruitment of keystone species from the rare subcommunity, rather than relying on the influx of new microbial species [57].

### 4.4. Community Assembly Processes

Our results revealed that stochastic processes dominated the assembly of both bacterial and fungal communities across all three regions (*AT*, *RT*, and *IT*) of Dianchi Lake. While this aligns with the view that stochasticity broadly shapes microbial assemblages [63], a clear shift was observed: the relative influence of deterministic processes was significantly higher for *RT* and *IT* compared to *AT*. This pattern likely reflects differences in ecological niches. The distributions of abundant taxa, often characterized by broader environmental tolerance, may be shaped more by random dispersal and drift [63]. In contrast, rare and intermediate taxa are often more sensitive to local environmental heterogeneity. Their survival and proliferation are often contingent on specific ecological conditions, rendering them more susceptible to deterministic filters such as environmental selection [63,64]. Spatially, this deterministic signal was strongest in the submerged macrophyte remnant area (*SubmP*), where the contribution of deterministic processes to *RT* and *IT* assembly was significantly greater than in the cyanobacterial bloom zone (*HABs*) or the heavily polluted Caohai area (*hPollut*). A possible explanation lies in the distinct environmental conditions of the *SubmP* area. The decomposition of plant litter releases a complex mixture of organic compounds and allelochemicals, creating a highly heterogeneous habitat [47]. Such biochemical complexity likely imposes stronger selective pressures on microbial metabolic capabilities, thereby reinforcing deterministic assembly. In our study, although both the *HABs* and *hPollut* sites exhibited extreme conditions, their stress regimes may have been comparatively uniform (e.g., high levels of nutrients or algal toxins), potentially resulting in weaker selective filtering compared to the multifaceted biochemical gradients present in the *SubmP* zone. These findings suggest that microbial community assembly in this lake ecosystem is governed by a balance of forces: while stochastic processes provide baseline control, deterministic selection plays a crucial, habitat-specific role in shaping the distribution of rare and intermediate taxa. This highlights the importance of considering both widespread stochasticity and localized deterministic enhancement when assessing microbial dynamics and function in freshwater environments.

### 4.5. Functional Differentiation and Environmental Adaptation of Microbial Communities

The predicted functional profiles of the microbial communities across the three regions displayed clear divergence, which was closely associated with the examined physicochemical properties and nutrient status, reflecting adaptive functional adjustment. The *hPollut* region exhibited typical eutrophication-associated features, characterized by the enrichment of toxin-producing cyanobacteria such as *Microcystis* PCC-7914 (22.6% relative abundance). This indicates a high risk of algal blooms and endogenous pollution pressure, as *Microcystis* can release microcystins, which pose severe ecological and public health risks [65]. Nitrogen-metabolizing groups (e.g., nitrogen fixation, urea hydrolysis) and organic pollutant degraders were also enriched. High nutrient levels provide sufficient substrates for urea-degrading microbes, accelerating nitrogen cycling [66]. Meanwhile, algal senescence promotes dissolved organic matter release, stimulating heterotrophic bacteria such as *Pseudomonas*, leading to a typical “bloom–decay” feedback loop [67]. In the *HABs* region, functional traits were centered around carbon cycling and phototrophy. For example, the abundance of cyanobacteria, such as *Planktothrix_NIVA-CYA_15*, was higher in the lake’s freshwater areas uninhabited by *Trapa japonica* compared to water inhabited by *T. japonica*, likely due to changes in environmental parameters and competition for sunlight, accompanied by the enrichment of methanotrophs and anoxygenic photoautotrophs [68]. Representative taxa such as *Methylosinus* and *Rhodobacter* utilize methane and reducing sulfur compounds as electron or energy sources, supporting high productivity under moderate nutrient conditions [69]. In contrast, *SubmP* displayed functional traits related to pollutant attenuation and metal cycling. Under low-oxygen to anaerobic conditions, *Flavobacterium* contributed to denitrification and organic pollutant degradation, consistent with a lower organic load [70]. Functional groups involved in dark sulfide oxidation, plastic degradation, and sulfate reduction were also enriched, suggesting active pollutant transformation and metal cycling. Fungal functional prediction indicated the dominance of saprotrophs in this relatively clean region, implying the recovery of decomposer communities and enhanced carbon turnover [71]. Nevertheless, the functional differentiation reported here was derived from predictive annotation tools (FAPROTAX and FUNGuild). Definitive functional confirmation will require further experimental approaches, with a particular emphasis on culturomics and integrative omics–AI strategies to bridge this gap [72].

## 5. Conclusions

This study provides a comprehensive assessment of the microbial diversity, assembly mechanisms, and ecological differentiation across distinct ecological zones of Dianchi Lake. Pronounced spatial heterogeneity was observed among the submerged macrophyte remnant area zone (*SubmP*), the heavily polluted Caohai area (*hPollut*), and the cyanobacterial bloom area (*HABs*), reflecting strong environmental filtering under eutrophic stress gradients. Total nitrogen and dissolved oxygen emerged as the primary environmental drivers shaping both the taxonomic composition and predicted functional structure. A key finding is the decoupling between richness and abundance across microbial communities. Rare and intermediate taxa accounted for the majority of taxonomic richness and exhibited significantly higher network robustness than abundant taxa, indicating their disproportionate contributions to community stability. Although stochastic processes dominated community assembly overall, deterministic selection played a greater role in structuring intermediate and rare taxa, particularly in the heterogeneous habitat of *SubmP*. These results suggest that ecological importance in eutrophic lake systems is not necessarily linked to numerical dominance but rather to functional diversity and interaction resilience. Functional differentiation among regions further revealed adaptive strategies under contrasting nutrient and habitat conditions, including enhanced nitrogen cycling in heavily polluted waters, increased phototrophic potential in bloom-dominated zones, and a stronger capacity for pollutant degradation in vegetated areas. Overall, this study highlights the critical ecological roles of rare and intermediate taxa in maintaining microbial network stability and spatial differentiation in eutrophic lakes. Effective lake management should therefore extend beyond controlling the dominant bloom-forming populations and prioritize nitrogen reduction, dissolved oxygen regulation, and the restoration of submerged vegetation to ensure sustained microbial-mediated ecosystem resilience.

## Figures and Tables

**Table 1:** Physicochemical properties of water in three representative areas of Dianchi Lake: the heavily polluted Caohai area (*hPollut*), the cyanobacterial bloom zone (*HABs*), and the submerged macrophyte remnant area (*SubmP*).

Water Property	Water Quality Limit ^†^			Sampling Site		
	Class III	Class IV	Class V	*hPollut*	*HABs*	*SubmP*
BOD_5_ (mg/L)	4	6	10	4.1 ± 0.2 a	3.9 ± 0.3 ab	3.64 ± 0.21 a
COD (mg/L)	20	30	40	36.2 ± 1.9 b	42.4 ± 2.2 a	42.5 ± 1.14 a
Total P (mg/L)	0.05	0.1	0.2	0.13 ± 0.01 a	0.12 ± 0.01 ab	0.10 ± 0.01 b
Total N (mg/L)	1	1.5	2.0	3.2 ± 0.11 a	2.1 ± 0.1 b	1.90 ± 0.06 c
SS (mg/L)	/	/	/	29.1 ± 3.3 a	23 ± 1.67 b	22.56 ± 1.45 b
Chlorophyll-a (μg/L)	/	/	/	94.1 ± 11.1 a	82.5 ± 7.5 ab	65.9 ± 4.5 b
Permanganate index (mg/L)	6	10	15	8.1 ± 0.5 b	9.4 ± 0.25 a	9.16 ± 0.21 a
pH	/	/	/	9.02 ± 0.1 a	9.08 ± 0.1 a	9.02 ± 0.07 a
Dissolved oxygen (mg/L)	5	3	2	10.7 ± 0.3 a	9.34 ± 0.32 b	8.63 ± 0.35 c
Water temperature (°C)	/	/	/	23.9 ± 0.4 a	23.01 ± 0.29 ab	22.69 ± 0.28 b
Transparency (m)	/	/	/	43.4 ± 3.5 a	43.17 ± 2.27 a	45.89 ± 1.8 a
Electrical conductivity (μS/cm)	/	/	/	485.9 ± 11.8 b	524.58 ± 2.77 a	528.78 ± 1.78 a

^†^ Limit set by the “China Surface Water Environmental Quality Standards” (GB3838-2002). (Means within the same row followed by different lowercase letters (a–c) indicate significant differences (one-way ANOVA, *p* < 0.05)).

## Data Availability

The raw data supporting the conclusions of this article will be made available by the authors on request.

## Supplementary Materials

The following supporting information can be downloaded at: https://www.mdpi.com/article/10.3390/microorganisms14030554/s1, Table S1. Topological properties of bacterial and fungal community co-occurrence networks in water samples from three representative regions of Dianchi Lake: *hPollut*, *HABs*, and *SubmP*; Table S2. Indicator species of bacterial and fungal OTUs in water samples from three representative regions of Dianchi Lake: *hPollut*, *HABs*, and *SubmP*.

## Abbreviations

The following abbreviations are used in this manuscript:

*SubmP*	Submerged macrophyte remnant zone
*hPollut*	Heavily polluted Caohai area
*HABs*	Cyanobacterial bloom zone
COD	Chemical oxygen demand
ACC	A chlorophyll-c
TN	Total nitrogen
TP	Total phosphorus
Chl-a	Chlorophyll-a
SS	Suspended substances
BOD_5_	Biochemical oxygen demand after 5 days
FLASH	Fast Length Adjustment of Short Reads
UPARSE	Uclust-Based Parser for ASV and OTU Clustering
RDP	Ribosomal Database Project
*AT*	Abundant taxa
*IT*	Intermediate taxa
*RT*	Rare taxa
βNTI	β-Nearest taxon index
RCbray	Raup–Crick based on Bray–Curtis
ANOVA	One-way analysis of variance
DO	Dissolved oxygen
