# Supplementary material for: Diversity, Assembly, and Habitat-Driven Dynamics of Microbial Communities in Eutrophic Dianchi Lake, Southwest China
Source: Microorganisms. 2026 Feb 28;14(3):554. doi: 10.3390/microorganisms14030554 (PMC13028956; doi:10.3390/microorganisms14030554)
Supplement: Supplementary file 1 [file microorganisms-14-00554-s001.zip › microorganisms-4137791-supplementary.pdf]

Supplementary materials

Table S1. Topological properties of bacterial and fungal community co-occurrence networks in water samples from three representative regions of Dianchi Lake: *hPollut*, *HABs*, and *SubmP*.

Samples	Nodes	Edges	Avg. degree	Modularity	Positive correlation ratio	Negative correlation ratio
hPollut	135	936	10.636	0.317	54.27%	45.73%
HABs	167	2279	23.617	0.272	55.2%	44.8%
SubmP	142	1445	15.622	0.231	53.77%	46.23%

Table S2. Indicator species of bacterial and fungal OTUs in water samples from the three representative regions of Dianchi Lake: *hPollut*, *HABs*, and *SubmP*.

Samples	Indicator species (Top10)			
	ID	Genus	Indicator	*P* value
**Bacteria**				
hPollut (126)	OTU9740	unclassified_o__Cytophagales	1	0.001
	OTU29142	*Elstera*	0.997992	0.001
	OTU24892	norank_f__NS9_marine_group	0.991304	0.001
	OTU4267	unclassified_c__Alphaproteobacteria	0.988787	0.001
	OTU8495	*Legionella*	0.988327	0.001
	OTU6406	*Flavobacterium*	0.985222	0.001
	OTU8506	unclassified_p__Pseudomonadota	0.980892	0.001
	OTU24975	*Ferruginibacter*	0.978208	0.001
	OTU733	*Gemmatimonas*	0.969325	0.001
	OTU4272	norank_o__SM1A07	0.966543	0.001
HABs	OTU12349	*Luteimonas*	0.706679	0.002
SubmP	OTU23732	*Acinetobacter*	0.896354	0.001
	OTU23993	*Acinetobacter*	0.874064	0.002
	OTU3506	*FukuN57*	0.757143	0.001
	OTU2051	norank_f__37-13	0.720721	0.001
	OTU3239	*Ephemeropptericola*	0.703704	0.001
	OTU23783	hgcI_clade	0.700071	0.003
**Fungi**				
hPollut (47)	OTU334	unclassified_k__Fungi	1	0.001
	OTU946	unclassified_k__Fungi	1	0.001
	OTU1027	unclassified_p__Chytridiomycota	1	0.001
	OTU1043	unclassified_k__Fungi	1	0.001
	OTU403	unclassified_k__Fungi	0.99815	0.001
	OTU311	unclassified_k__Fungi	0.997489	0.001
	OTU1018	unclassified_p__Chytridiomycota	0.992758	0.001
	OTU13306	unclassified_p__Chytridiomycota	0.992715	0.001
	OTU362	unclassified_p__Chytridiomycota	0.992346	0.001
	OTU17819	unclassified_k__Fungi	0.980711	0.001
HABs	/	/	/	/
SubmP	OTU13301	*Metschnikowia*	0.753706	0.038
